# Color Centers in BaFBr Crystals: Experimental Study and Theoretical Modeling

**DOI:** 10.3390/ma17133340

**Published:** 2024-07-05

**Authors:** Talgat Inerbaev, Abdirash Akilbekov, Daurzhan Kenbayev, Alma Dauletbekova, Alexey Shalaev, Elena Polisadova, Marina Konuhova, Sergei Piskunov, Anatoli I. Popov

**Affiliations:** 1 Department of Technical Physics, L.N. Gumilyov Eurasian National University, Satpayev Str. 2, Astana 010008, Kazakhstan; talgat.inerbaev@gmail.com (T.I.); akilbekov_at@enu.kz (A.A.); daurzhankenbayev@gmail.com (D.K.); ak.daukletbekova@gmail.com (A.D.); 2 Vernadsky Institute of Geochemistry and Analytical Chemistry, Russian Academy of Science, 119991 Moscow, Russia; 3 Vinogradov Institute of Geochemistry SB RAS, Favorskii Str. 1a, 664033 Irkutsk, Russia; alshal@igc.irk.ru; 4 School of Advanced Manufacturing Technologies, National Tomsk Polytechnic University, 634050 Tomsk, Russia; polisadova72@gmail.com; 5 Institute of Solid State Physics, University of Latvia, Kengaraga 8, LV-1063 Riga, Latvia; marina.konuhova@cfi.lu

**Keywords:** single crystal BaFBr, swift heavy ions, optical absorption, color centers, density functional theory (DFT), VASP

## Abstract

This study presents theoretical and experimental investigations into the electron and hole color centers in BaFBr crystals, characterizing their electronic and optical properties. Stoichiometric BaFBr crystals grown by the Steber method were used in the experiments. Radiation defects in BaFBr crystals were created by irradiation with 147 MeV ^84^Kr ions with up to fluences of 10^10^–10^14^ ions/cm^2^. The formation of electron color centers (F(F^−^), F_2_(F^−^), F_2_(Br^−^)) and hole aggregates was experimentally established by optical absorption spectroscopy. Performed measurements are compared with theoretical calculations. It allows us to determine the electron transition mechanisms and investigate the processes involved in photoluminescence emission in Eu-doped BaFBr materials to enhance the understanding of the fundamental electronic structure and properties of electron and hole color centers formed in BaFBr crystals.

## 1. Introduction

Significant progress has been made in the development and comprehensive use of radiation detectors based on alkaline earth fluorides, such as BaFBr doped with europium, cerium and other dopants, which are commonly used as an imaging plate (IP) [1,2,3,4,5]. BaFBr-Eu^2+^ crystals can store energy when absorbed by X-rays in the form of electron-hole pairs at metastable centers, which can be used for stable imaging at room temperature [6,7,8,9,10,11,12,13,14,15,16]. This technology has been successfully used in medicine and biology for digital radiography, allowing the radiation dose to patients to be significantly reduced. Commercially marketed IP typically contains BaFBr-Eu^2+^ powder in an organic binder and demonstrates high efficiency for stored X-ray energy. However, the microstructure of hole storage centers and the processes associated with photoluminescence emission are not yet fully understood. Electronic color centers containing electrons play a decisive role in these processes. The nature of such F-type electronic centers, namely electrons captured in fluorine and bromine vacancies, as well as electronic aggregate color centers, has been studied experimentally in sufficient detail, while hole color centers have not yet been fully identified.

It is worth noting that BaFBr single crystals can be easily cleaved into thin layers. In Ref. [17], the authors used density functional theory (DFT) to analyze BaFBr monolayers and determine their optical properties, such as absorption coefficient, conductivity, refractive index, and dielectric function. According to the study [17], these monolayers appear transparent above 25 eV and exhibit a high absorption coefficient in the range of 10–20 eV. Due to their efficient light emission and absorption capabilities, they hold potential for use in future nano- and ultrathin optoelectronics.

Previous research has focused on the characteristics of intrinsic defects like F-centers and vacancies at fluorine and bromine sites, as well as the aggregation of two F-centers in BaFBr. However, up to now there has been limited detailed study on complementary hole centers. A thorough understanding of the electronic structure and properties of intrinsic point defects in BaFBr crystals is essential for improving their performance in various applications, including image plates and other image plate devices. Understanding the formation and behavior of these defects provides valuable insights into the electronic structure and defect physics of BaFBr crystals. Additionally, theoretical calculations, such as DFT simulations, help in interpreting the experimental results and provide a deeper understanding of the nature of the optical absorption spectra observed. This study presents theoretical and experimental investigations into the electron and hole color centers in BaFBr crystals. It characterizes their optical properties, such as absorption coefficients, using DFT. Our study aims to determine the electron trapping mechanisms and investigate the processes involved in luminescence processes in BaFBr materials, with the ultimate goal of enhancing our understanding of the fundamental electronic structure and properties of electron and hole color centers in BaFBr crystals.

## 2. Computational Details

Theoretical studies using density functional theory have been widely employed to investigate the electronic structure and properties of defects in various materials, including insulating crystals like BaFBr. The ability of DFT to provide detailed insights into the energetics and electronic behavior of defects has made it a powerful tool for understanding and predicting the properties of color centers in crystals like BaFBr. A self-consistent solution of the Kohn–Sham equations, as implemented in the Vienna ab initio simulation package (VASP) [18,19], is employed to determine the electronic structure using DFT. This takes into account Coulomb correlation and exchange electron–electron interactions, as well as electron–ion interaction. In this study, both the hybrid functional Heyd–Scuseria–Ernzerhof (HSE06) [20] and the Perdew–Burke–Ernzerhof (PBE) [21] exchange–correlation functional under the generalized gradient approximation (GGA) were used for comparison purposes. The calculations utilized projector-augmented-wave formalism [22] based on a pseudopotentials approach on a plane-wave basis provided by VASP. Ground state electronic structure calculations using HSE06 yielded more precise band gap energies compared to those obtained with PBE. However, PBE is computationally more efficient when it employs the GGA exchange–correlation functional.

BaFBr crystals have a tetragonal structure of PbFCl (space group #129, P4/nmm) [23]. For defect simulations, 2 × 2 × 2 supercells comprising a total of 48 atoms were utilized. The lattice defects were simulated by generating charge-occupied vacancies at F or Br positions (F-centers) and double F or Br charge-occupied vacancies at the nearest-neighboring positions in model supercells (F_2_ centers). These defect simulations were performed to explore the energetics, electronic and optical properties of the color centers in BaFBr crystals.

The geometry optimization procedure involved minimizing stresses and forces while simultaneously allowing the shape and volume of the unit cell to relax. This was achieved using the GGA PBE method, with an initial pre-optimization of geometry through GGA PBE before conducting HSE06 calculations to expedite the optimization procedure. The application of the GGA PBE exchange–correlation functional made use of a conventional Monkhorst-Pack grid with a 4 × 4 × 2 sampling k-mesh, while for HSE06 calculations, a 2 × 2 × 1 sampling k-mesh at the Γ-point-centered grid was used. In addition, computations were performed utilizing the many-body perturbation theory in the GW approach to analyze the electronic properties of perfect BaFBr lattice [24]. A single unit cell with 6 × 6 × 4 sampling k-mesh was employed in this case. By comparing the results of GW and HSE06 calculations to experimental data, the error introduced by the hybrid functional during the simulation can be estimated. A threshold energy of 500 eV was employed in all computations, alongside a spin-unrestricted approach to derive each electronic structure. Visualization of outcomes was carried out using the VESTA program [25].

The optical properties of F- and H-centers in BaFBr crystals were analyzed by means of the transition dipole moment matrix elements:

(1) $$D_{\sigma ,ij}=e\left\langle \psi_{\sigma ,i}^{KS}|r|\psi_{\sigma ,j}^{KS}\right\rangle .$$

For transitions between the initial state σ, *i* and final state σ, *j* calculated on the basis of Kohn−Sham orbitals $\psi_{\sigma ,i}^{KS}$, where σ is a spin index, and *e* is an elementary charge. The transition dipole moment was used for calculating the oscillator strength:

(2) $$f_{\sigma ,ij}=\frac{4\pi m\nu_{\sigma ,ij}}{3e^{2}\hbar }{\left|D_{\sigma ,ij}\right|}^{2},$$

where *m* and *ħ* are the electron mass and Plank constant, respectively, and ν_σ,ij_ is the frequency of transition between the *i*th and *j*th states. Using the oscillator strengths and neglecting spin–orbit coupling, the absorption spectra can then be determined as a(ν) = a_α_(ν) + a_β_(ν), where $\alpha_{\sigma }\left(\nu \right)=\sum_{ij}f_{\sigma ,ij}\delta (\nu -v_{\sigma ,ij})$.

## 3. Experimental Setup

Experimentally studied BaFBr crystals were grown according to the Shteber method on a special device (Irkutsk, Russia) in a graphite crucible in a helium-fluoride atmosphere using stoichiometric mixtures of BaBr_2_ and BaF_2_. The BaFBr crystal samples were irradiated with 147 MeV and ^84^Kr ions at 300 K with fluences ranging from 10^10^ to 10^14^ ion/cm^2^. Optical absorption spectra were measured at room temperature on a two-beam spectrophotometer UVI-VIS SPECORD 250 PLUS (Jena, Germany).

BaFBr crystal samples were irradiated with 147 MeV ^84^Kr ions at 300 K to fluences (10^10^–10^14^) ion/cm^2^ at the DC-60 heavy ion accelerator (Astana, Kazakhstan). The sample temperature during irradiation was about 70–90 C. Plate samples prepared for irradiation were 10–12 mm long, 9–10 mm wide, and about 1 mm thick.

The electronic and nuclear energy loss analysis of 147 MeV ^84^Kr ion in BaFBr crystal using SRIM [26] is shown in Table 1.

## 4. Results and Discussion

Ionization-specific energy losses (electronic losses) exceed the energy loss due to elastic collisions (nuclear losses). The ratio between them is 10:1. Thus, the mechanism of electronic excitations dominates, but not so significantly, so the contribution of nuclear energy losses must be taken into account.

The processes behind the creation of defects in BaFBr crystals were primarily studied with europium-activated crystals. This research provided insight into the photoluminescence (PL) of europium ions. A model reported in Refs. [27,28] was introduced, assuming the presence of $v_{Br}$ (Br vacancies) in the initial crystalline lattice. The irradiation of crystals by X-rays leads to the generation of e and h (free electrons and holes). Electrons are captured by $v_{Br}$ and thus F(Br)-centers are formed. Simultaneously, holes are captured by Eu^2+^, transforming it into Eu^3+^. Photoexcitation of F(Br)-centers leads to the excitation of electrons to the conduction band (CB) and recombination with holes on the europium ion. Recombination leads to an excited state of the europium ion, which relaxes, emitting a characteristic 390 nm luminescence.

By analogy with the alkali-halide crystals [10,29], as suggested for Eu^2+^-doped BaFBr crystals in Ref. [30], X-rays generate excitons. After that, already self-trapped excitons are decayed with the creation of a Frenkel pair: F(Br^−^) + H-center, where H-center is an interstitial bromine atom (${Br}_{2}^{-}$). Upon photostimulation, the electron released from the F-center can recombine with the H-center. The recombination energy is transferred to excite Eu^2+^, leading to PL. Studies on photoluminescence (PL) in BaFBr:Eu have not yet detected an increase in Eu^3+^ concentration or H-centers following X-ray irradiation. Due to the absence of experimental verification of the previous two models, a third model was developed in [31,32,33]. It has been demonstrated that oxygen is consistently present in BaFBr, regardless of the method used to prepare it, as an unregulated impurity. Oxygen replaces fluoride ions forming $O_{F}^{2}$ centers involving Br-vacancy ($O_{F}^{2}$ + $v_{Br}$). X-rays have been shown to generate F(Br^−^) + V_k_ pairs in BaFBr single crystals, where V_k_ is a self-trapped hole. Recombination of an electron from F(Br) with the V_k_ center releases energy that excites the luminescence of europium. Although the V_k_ center is yet spectroscopically unidentified, this model is not contradicted by the experiment. The formation of spatially correlated ternary aggregates of F, V_k_ and Eu^2+^ has been suggested elsewhere [10,31,34].

**Figure 1:**
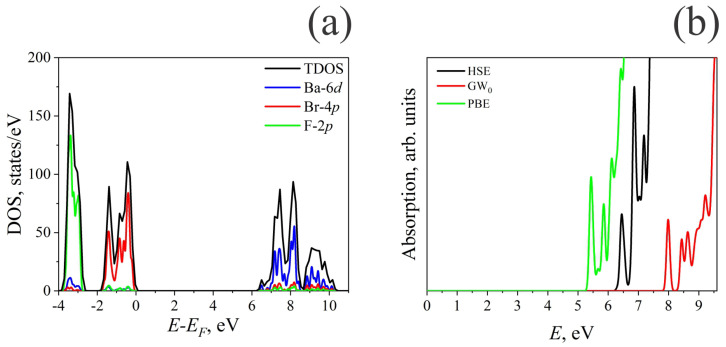
Electronic properties of perfect BaFBr lattice: (**a**) HSE06 calculated projected density of electronic states; (**b**) HSE06, *GW*_0_, and PBE calculated optical absorption spectra.

Before modeling the effect of defects on the optical properties of the compound under study, we studied the electronic properties of a perfect BaFBr crystal. The calculations for the density of electronic states (DOS) and optical absorption spectra (PBE, HSE06, and GW0 calculations) are shown in Figure 1a. The results of the DOS calculations are qualitatively the same, so only the results of the HSE06 calculations are presented here.

As seen in Figure 1a, the Ba-6*d* states form the conduction band of the BaFBr crystal. The O-2*p* states form the band from −1.7 eV to the Fermi level, while the F-2*p* states are responsible for the band from −3.8 to −2.7 eV below the Fermi level. Thus, in a BaFBr crystal at relatively low energies, absorption of photons occurs due to O-2*p*→Ba-6*d* transitions, while with increasing radiation energy, absorption due to F-2*p*→Ba-6*d* transitions is possible.

Figure 1b shows the values of linear optical absorption calculated by the PBE, HSE, and GW0 methods. Experimental values of the threshold of the excitation spectrum were measured to be equal to 7.6 eV [35], while other studies report a value of this value equal to 8.3 eV [27,36]. In particular, in Ref. [35], the presence of two absorption peaks due to the so-called halogen doublet was noted. The second peak corresponds to an energy of 8.1 eV. The calculated absorption threshold values are 5.4, 6.4, and 8.0 eV for the PBE, HSE, and *GW*_0_ calculations, while the second absorption peaks are located at energies of 5.8, 6.9, and 8.4 eV, respectively.

The *GW*_0_ simulation results agree much better with the experimental data than other simulation methods. The most sizable error is the band gap derived with a PBE functional. Using the hybrid HSE06 functional improves the agreement between theory and experiment, but the error remains quite large. This is likely because the values of the Hartree–Fock mixing parameter and the screening parameter in the case of BaFBr do not work correctly and should be revised. Such a study was carried out in the case of oxides in Ref. [37], where it is shown that a virtually infinite number of combinations of the two parameters could reproduce the experimental band gap. However, no single pair can describe the entire set of materials examined. The results, on the other hand, show that it is possible to calculate these materials’ electronic structure using a functional model that includes shielded exact exchange and the correlation contribution that goes with it. Fitting these parameters to adequately describe the electronic properties of BaFBr using the hybrid functional is beyond the scope of this study, while the GW approach is too computationally expensive. For these reasons, the results below for the electronic properties of defective crystals were obtained using the HSE06 functional. The above data for a perfect lattice allows us to estimate the possible error of the data received.

Computer modeling of F- (halide vacancy), F_2_- (double halide vacancy), and I-centers (interstitial halide ions) in BaFBr crystals leads to a deeper understanding of its electronic and optical properties. Table 2 summarizes the results of optical transition properties calculations in a BaFBr crystal containing the defects considered in this work. It shows the oscillator strength *f* and transition energy Δ*E* values, as well as the electronic orbitals involved in bright optical transitions for the α (spin-up) and β (spin-down) states with f > 0.1.

**Figure 2:**
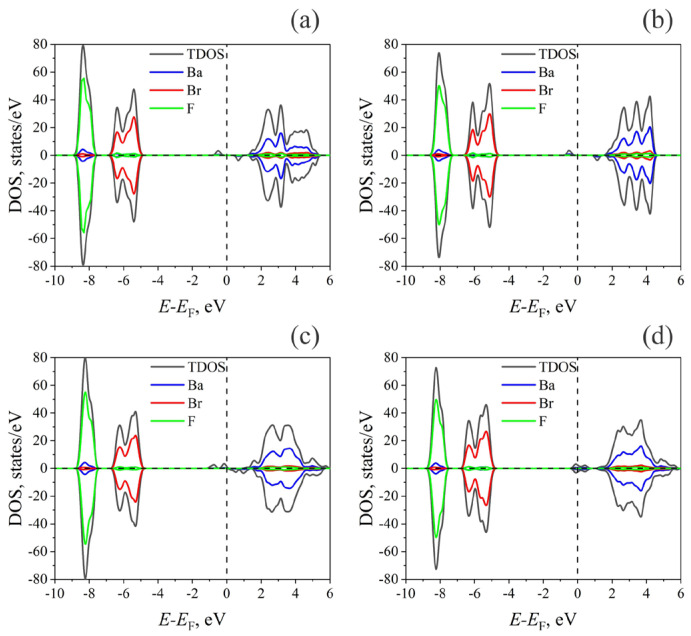
HSE06 calculated total density of states (TDOS) and projected density of electronic states (PDOS) for defective BaFBr 2 × 2 × 2 supercells (**a**) F(Br) center, (**b**) F(F) center, (**c**) F_2_(Br^−^) center, (**d**) F_2_ (F) center. Dashed line corresponds to the Fermi level.

The results of the influence of these defects on the density of electronic states of BaFBr are illustrated in Figure 2.

The electronic structure of the valence band (VB) and CB mainly does not change after the creation of F- and F_2_-centers in BaFBr and coincides with perfect crystal (Figure 2). In the case of single F-centers at the Br (Figure 2a) and F (Figure 2b) positions, additional filled states with spin-up below the Fermi level appear in the band gap of BaFBr, and with spin-down, above the Fermi level. In the case of F_2_ centers at the Br position (Figure 2c), the situation is similar, except that now, in the band gap, there are two peaks for filled states with spin-up and two peaks for empty states with spin-down. (Figure 2c) For an F_2_ center at F nodes, each spin-up peak corresponds to a spin-down peak both above and below the Fermi level (Figure 2d).

**Figure 3:**
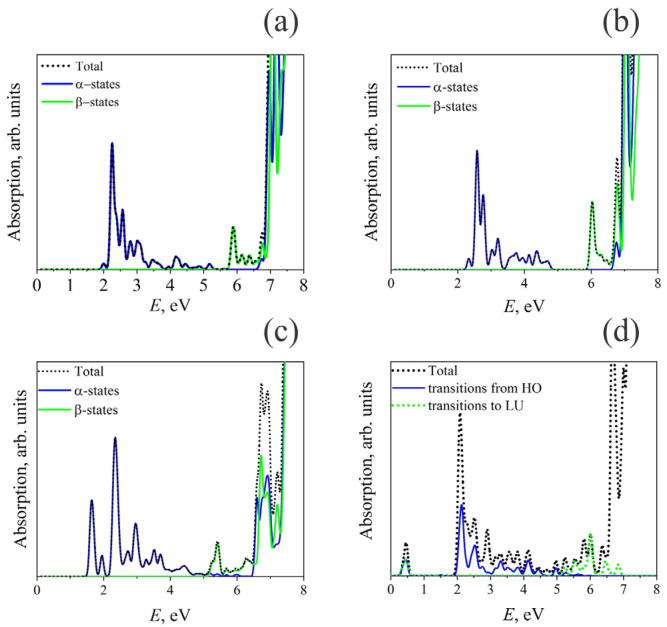
HSE06 calculated optical absorption spectra for BaFBr crystals containing F- and H-centers: (**a**) F(Br^−^) center, (**b**) F(F^−^) center, (**c**) F_2_(Br^−^) center, (**d**) F_2_(F^−^) center.

Changes in the electronic DOS caused by defects lead to significant changes in the optical absorption spectra. Figure 3 shows the calculated absorption spectra for BaFBr crystals containing F- and F_2_-centers.

For all the considered cases of defects of the F and F_2_ types, the reasons for the change in the optical absorption spectrum compared to a perfect crystal are qualitatively the same. The differences are caused by the number of defective electronic levels and their relative energies. Figure 5 presents a diagram of all considered electronic transitions responsible for optical absorption in the considered systems.

The partial charge densities for electronic states that take part in bright optical transitions in BaFBr with a defect of the F(Br) center type are shown in Figure 3. The band of F(Br^−^)-center that possesses the highest oscillator strength f = 1.094, which indicates the strongest optical transition, has a maximum energy level of E_max_ = 2.25 eV. The other two bands have energies of E = 2.38 eV with an oscillator strength of f = 0.426 and E = 2.52 eV with an oscillator strength of f = 0.4904. These predictions are in agreement with the absorption spectra yielding experimentally identified absorption bands for F(Br) centers at 2.15 eV [38], 2.14 eV (γ-ray irradiation at RT, T_meas_ = 290 K) [39], 2.15 eV (X-ray at RT, T_meas_ = 10 K) [31], and 2.18 eV (X-ray at RT, T_meas_ = 290 K) [40].

**Figure 4:**
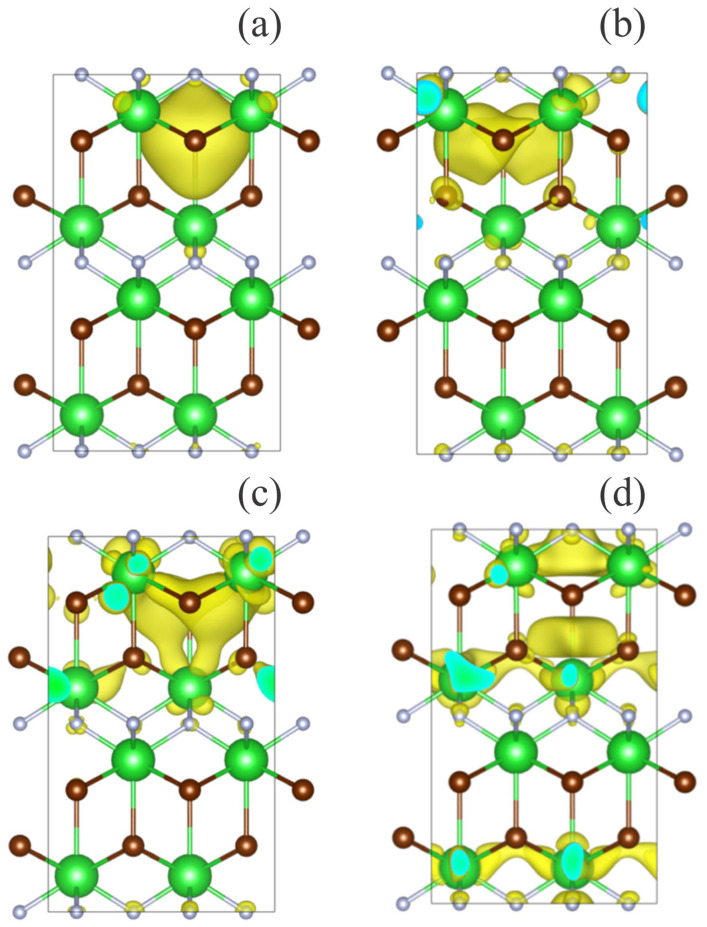
Visualization of the space distribution of electron densities for spin-up states that create the brightest optical transitions in a BaFBr crystal with an F(Br^−^)-center (Table 2). (**a**) HO (#189), (**b**) LU + 1 (#191), (**c**) LU + 2 (#192), and (**d**) LU + 4 (#194). Green balls stand for Ba atoms, white balls stand for F atoms, while brown balls stand for Br atoms.

Analysis of the absorption spectrum calculated for F(Br)-center in BaFBr crystal (Figure 3a) shows that the strongest optical transitions are observed for states of spin-up electrons from the HO level to the LU + 1, LU + 2, and LU + 4 levels. Obviously, spin-down electrons play a minor role in optical transitions, having an oscillator strength close to the threshold of 0.1. This prediction is supported by the calculated total and projected density of state (TDOS and PDOS) shown in Figure 2. The states mentioned above relate to the F-center and can be observed through the electron density distribution (Figure 4).

The HO and LU + 1 states have high electron density around the empty Br site (Figure 2a,b), while the LU + 2 and LU + 4 states have partial filling of Ba 6*d* levels (Figure 4c,d). The presence of F(Br)-center in BaFBr results in the appearance of donor level at 2.0 eV below the CB minimum and acceptor level at 5.7 eV above the VB maximum (Figure 4a). Because of this, optical absorption starts in this system at 2 eV due to transitions from the F(Br) center spin-up state to the Br-6d orbitals of the conduction band minimum. Absorption starting at an energy of 5.7 eV corresponds to transitions from VB to an unoccupied defect energy level. Further absorption is caused by O-2*p*→Ba-6*d* transitions as it is for the host BaFBr lattice.

**Figure 5:**
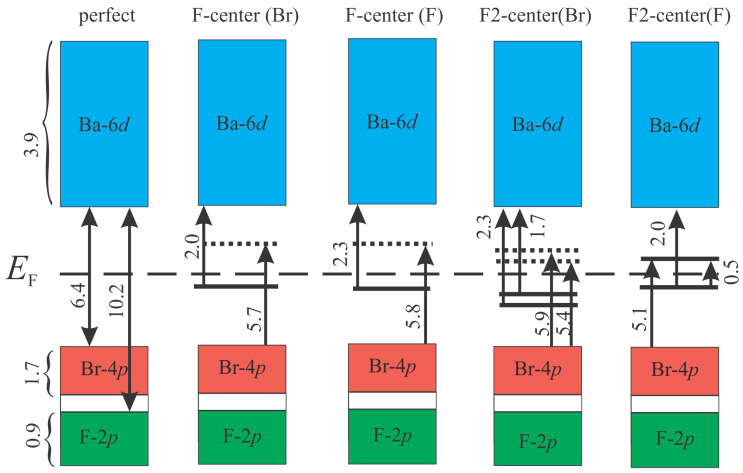
Schematic illustration of HSE06 calculated band structure and optical transitions in BaFBr containing the defects of F- and F2-center types. The numbers show the widths of electronic bands and the threshold values for the onset of optical absorption in eV. Solid lines correspond to the electronic levels of α states, while dotted lines indicate β states. The dashed line represents the Fermi level.

The electronic structure calculated for the F(F)-center in BaFBr (Figure 2b) is similar to the F(Br)-center. The only difference is that in the case of F(F)-center, an occupied defect-induced level is located at 2.3 eV below the CB minimum and an unoccupied one at 5.8 eV above the VB maximum (Figure 2b). Optical absorption spectra calculated for the F(F)-center in BaFBr (Figure 5b) are similar to the optical absorption spectra predicted for F(Br)-center. Optical absorption happens in the energy ranges of 2.3 to 4.9 eV because of transitions from the F(F) center’s spin-up state to the conduction band. Absorption in the energy range 6–6.5 eV occurs due to excitations from valence band levels to an unoccupied F(F) center spin-down state.

**Figure 6:**
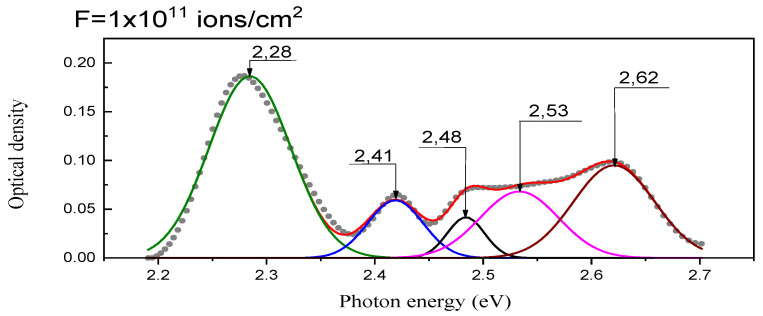
The absorption spectrum of BaFBr crystal irradiated with 147 MeV Kr ions with a fluence of 10^11^ ions/cm^2^.

The band with the maximum energy level of E_max_ = 2.57 eV has the highest oscillator strength, f = 0.687, indicating the brightest transition. The other two bands have energies of E = 2.75 eV with an oscillator strength of f = 0.468 and E = 2.606 eV with an oscillator strength of f = 0.441. The absorption spectra experimentally identified absorption bands for F(F^−^) centers at 2.50 eV [38], 2.72 eV (Ad. colored, T_meas_ = 290 K) [40], and 2.65 eV (X-ray at RT, T_meas_ = 10 K) [31]. Additionally, for BaFBr crystals irradiated with 147 MeV Kr ions, a band of 2.62 eV was observed and theoretically predicted (Figure 3 and Figure 6).

The optical absorption spectrum in the case of a double defect of the type F_2_(Br) center is presented in Figure 3c. In this case, two filled electronic levels appear in the band gap, corresponding to spin-up states. (Figure 1c) These levels are located at 1.7 and 2.3 eV below the CB minimum. Also, in this system, two unfilled spin-down states are now located at 5.4 and 5.9 eV above the VB maximum. As a result, transitions between spin-up states from occupied levels within the band gap to states in the conduction band cover the energy range from 1.7 to 6.2 eV. Transitions from O-2*p* VB states to acceptor defect levels in the band gap with spin-down, on the other hand, happen in the energy range of 5.4 to 7.6 eV. However, most electronic transitions have a low oscillator strength, which causes a relatively low intensity of optical absorption in the energy range of 3.2–5.3 eV.

The optical absorption spectrum in the case of a double defect of the type F_2_(F) center is presented in Figure 3d. The difference between the BaFBr structure with a double defect of the type F_2_(F) center is that the electron density of states becomes symmetrical concerning the spin direction. Now, inside the bandgap, there are a pair of spin-up and spin-down occupied and a pair of empty spin-up and spin-down defective levels in the bandgap. As a result, the following additional types of optical transitions are possible in the system compared to the perfect structure: (i) excitations from occupied defect levels to unoccupied defect levels, (ii) transitions from occupied defect levels to Ba-6*d* states of the conduction band, and (iii) transitions from O-2*p* levels of the valence band to unoccupied defect levels.

This system’s filled and unfilled defect levels are HO and LU orbitals, respectively. Transitions between levels of the F_2_(F) center occur at an energy of approximately 0.5 eV. Optical absorption due to transitions of HO orbitals to the Ba-6*d* conduction band states occurs in the energy range from 2 to 5.9 eV. However, in this case, the brightest transitions with a relatively large value of oscillator strengths occur upon excitation to states close to the bottom of the conduction band. As the photon energy increases, the number of transitions becomes more significant because the PDOS of the Ba-6*d* states is considerable. However, small values of the oscillator forces lead to the intensity of optical absorption decreases. Transitions from the valence band to LU states occur in the energy range from 5.1 to 6.8 eV, which corresponds to the width of the O-2*p* band. The presence of peaks in the absorption spectrum during these transitions is explained by the peculiarities of PDOS O-2*p* states and the dependence of the calculated oscillator strengths on the transition energy. The remaining optical absorption occurs due to electronic transitions in the host matrix of the BaFBr crystal.

In addition to the types of optical transitions considered, photon absorption is also possible due to F-2*p*→Ba-6*d* transitions. According to the results of HSE06 calculations, in the case of a perfect BaFBr crystal, absorption of this type is located at energies of 10.2 eV and higher. As follows from the data in Figure 2, the BaFBr crystal with a defect of the F_2_(F)-center type should have the lowest energy of such transitions, where the threshold value of such transitions should be 7.6 eV. However, this value is 1.2 eV higher than the optical absorption threshold of perfect crystal calculated using the HSE06 functional. For this reason, one cannot expect such transitions to introduce changes in the absorption spectrum of the compounds studied.

At temperatures above 200 K, F(F) centers are known to be generated, but the reason for this is not yet understood [41]. F(Br) centers are more dominant than F-centers at the fluorine positions in BaFCl and BaFBr materials. This is because the formation of fluorine ion vacancy is more energetically complicated than for bromine or chlorine vacancies, making vacancies more easily formed at the chlorine or bromine site than at the fluorine site. When stored in the dark at room temperature, F(Br^−^) centers disappear in X-ray irradiated BaFBr crystals, and the 1.35 eV absorption band associated with the F_2_ center increases significantly, while there is also a slight increase in F(F) [4,42]. When crystals are irradiated with increasing doses (fluence) of krypton ions from 10^13^–10^14^ ions/cm^2^, aggregate electronic color centers of F_2_-type are created. These centers are formed at long storage (after a week or more) at room temperature after the corresponding irradiation. The absorption bands for F_2_(Br^−^) centers are identified at 1.36 eV (X-ray irradiation at 290 K) [40] and also when irradiated with krypton ions (Figure 6). This optical absorption peak can be associated with the HO→LU + 1 transition for α-states and an energy of 1.68 eV (Table 2).

**Figure 7:**
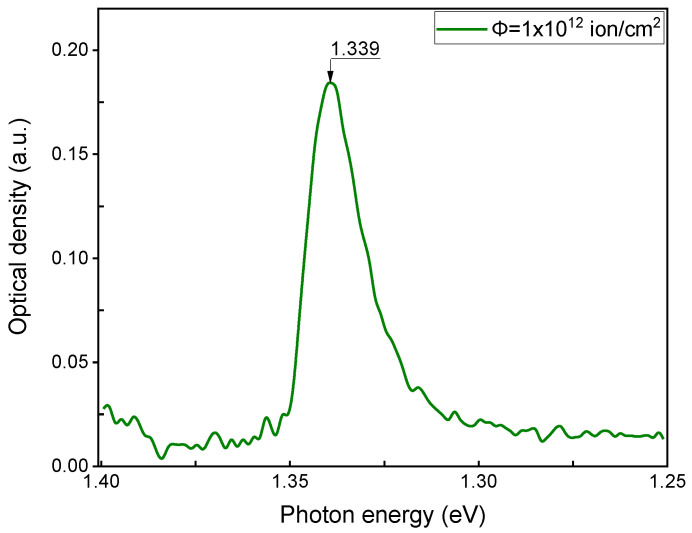
The absorption spectrum of BaFBr crystal irradiated with 147 MeV Kr ions with a fluence of 10^12^ ions/cm^2^.

The F_2_(F^−^) center has a calculated absorption maximum of 2.064 eV (Table 2 and Figure 3), the maximum of which is close to the absorption maximum of the F(Br) center. Note that the 2.28 eV band for BaFBr irradiated with krypton ions is closest to the absorption of the F_2_(F) center, Figure 7.

Complementary defects to F-centers are H-centers, which are well-studied in alkali halides [43,44,45,46]. Our experiments, as performed in [47], only indirectly confirm the creation of H-centers by the formation of ${Br}_{3}^{-}$- aggregate centers. In the Raman spectra of KBr crystals induced by V centers, bands at 175 cm^−1^, 265 cm^−1^, and 349 cm^−1^ (the first overtone of the 175 cm^−1^ mode) were determined [48]. For bromine compounds, Raman modes with frequencies around 175 cm^−1^ for ${Br}_{3}^{-}$ and 265 cm^−1^ for Br_2_-type centers have been observed, which encompass any polyhalide ion such as Br_5_ or even higher polyions. In the Raman spectra of BaFBr, irradiated with 147 MeV ^84^Kr ions, all these vibrational modes can be observed.

Before moving to conclusions, it is necessary to note that a comprehensive understanding of the mechanisms of point defect formation in BaFBr, their defect stabilization at different irradiation temperatures and subsequent effective recombination is absolutely important for the successful use of BaFBr and similar materials as a recording media in imaging plate detectors.

The nature of electron trap centers is well studied: these are mainly F-centers in fluorine and bromine sublattices (F(F^−^) and F(Br^−^)) [2]. Holes are believed to be localized near Eu^2+^ ions, but the microstructure of hole storage centers and the processes associated with the formation of PSL centers are still debated [2,27,28]. It is known that in BaFBr:Eu^2+^, the highest PSL signal can be achieved through photostimulation of F(Br^−^) color centers.

Therefore, among various formulations for the production of BaFBr, optimized with various additives, those in which the most preferable formation of F(Br^−^) centers will be realized and the formation of other electronic defects will be suppressed will be preferable. Note that in the case of irradiation with ion beams, the situation may be more complex because the mutual concentration of electron centers may depend on the type of ions as well as on their energy [49,50,51]. Note that a deep comparison of new photostimulated compounds with previously well-studied alkali halides and BaFBr always helps not only to understand the mechanisms but also to speed up their optimization [2,4,27,28,30,52,53,54,55,56,57,58,59,60,61].

## 5. Conclusions

This study has made a contribution to elucidating the complex interplay between radiation-induced defects and the optical properties of BaFBr crystals. Through a combination of a number of measurements and theoretical modeling, we have confirmed the formation of electron and hole color centers, notably F(Br), F(F), F_2_(F), and F_2_(Br). The agreement between density functional theory (DFT) calculations and optical absorption spectroscopy has been instrumental in interpreting the nature of predicted absorption spectra, thereby advancing our understanding of defect-induced optical phenomena in BaFBr crystals. The use of both PBE and HSE06 functionals, as well as *GW*_0_ calculations in our DFT calculations, has provided a framework for fast and accurate modeling of the defects and their impact on the crystal’s optical properties. Our study has successfully bridged the gap between theoretical predictions and experimental observations.

## Figures and Tables

**Table 1:** Radiation parameters of 147 MeV ^84^Kr ions in BaFBr.

Ion	Energy, MeV	(dE/dx)e, keV/nm	(dE/dx)n, keV/nm	R, µm
^84^Kr	147	12.04	1.36	17.87

**Table 2:** Electronic properties, such as position and oscillator strength of highest occupied (HO) and lowest unoccupied (LU) states in both spin-up (α states) and spin-down (β states) orientations for the optical transitions with oscillator strength *f* > 0.1 as calculated in this study.

Structure	Donor	Acceptor	Osc. Str.	ΔE, eV
F(Br) center	α states			
	HO	LU + 1	1.094	2.25
	HO	LU + 2	0.426	2.38
	HO	LU + 4	0.490	2.57
	β states			
	HO	LU	0.172	5.85
	HO-1	LU	0.114	5.90
F(F) center	α states			
	HO	LU + 1	0.687	2.57
	HO	LU + 2	0.441	2.61
	HO	LU + 4	0.469	2.75
	β states			
	HO	LU	0.325	6.01
F_2_(Br) center	α states			
	HO-1	LU + 5	0.329	2.94
	HO-1	LU + 6	0.467	2.99
	HO	LU + 1	0.675	1.68
	HO	LU + 3	0.732	1.95
	HO	LU + 12	0.488	2.33
	HO	LU + 13	0.457	2.35
	HO	LU + 15	0.285	2.49
	HO	LU + 18	0.250	2.66
	β states			
	HO	LU	0.227	5.25
	HO-1	LU	0.219	5.25
	HO-2	LU	0.574	5.37
	HO-3	LU	0.466	5.44
	HO-4	LU	0.368	5.44
F_2_(F) center	α states			
	HO	LU	0.467	0.45
	HO	LU + 3	1.038	2.06
	HO	LU + 5	0.415	2.16
	HO	LU + 10	0.221	2.41
	HO	LU + 14	0.594	2.51
	HO	LU + 33	0.208	3.14
	HO	LU + 43	0.111	3.64
	HO	LU + 47	0.101	3.83
	HO	LU + 54	0.112	4.05
	HO	LU + 56	0.119	4.10
	HO	LU + 58	0.133	4.18
	HO	LU + 64	0.202	4.96
	β states			
	HO	LU	0.152	0.37
	HO	LU + 5	0.746	2.14
	HO	LU + 7	0.185	2.26
	HO	LU + 10	0.119	2.43
	HO	LU + 15	0.191	2.56
	HO	LU + 27	0.125	3.27
	HO	LU + 30	0.113	3.34
I-center (Br^−^)	α states			
	HO-49	LU	0.151	5.00
I-center (F^−^)	α states			
	HO	LU	0.239	1.94
	HO-31	LU	0.154	3.16
	HO-32	LU	0.116	2.99
	HO-33	LU	0.637	3.03
	HO-27	LU	0.411	4.32

## Data Availability

Data is contained within the article. Additional data can be provided upon request.
